# Comparison of One-Year Outcomes in Sleeve Gastrectomy vs. One Anastomosis Gastric Bypass in a Single Bariatric Unit

**DOI:** 10.7759/cureus.74838

**Published:** 2024-11-30

**Authors:** Kieran Das, Faisal Nadeem, Syed A Kabir

**Affiliations:** 1 General Surgery, Walsall Manor Hospital, Walsall, GBR; 2 Upper GI Surgery, Lancashire Teaching Hospital NHS Foundation Trust, Preston, GBR; 3 General Surgery and Bariatric Surgery, Walsall Manor Hospital, Walsall, GBR; 4 Laparoscopic Surgery, Maroof International Hospital, Islamabad, PAK

**Keywords:** bariatric surgery complications, bariatric surgery mesh, bariatric surgery/therapeutic use, diabetes and bariatric surgery, minimal access bariatric and laparoscopic surgery

## Abstract

Introduction

Sleeve gastrectomy (SG) is the most popular bariatric procedure worldwide in terms of numbers performed. However, there has been a rise in the popularity of the one anastomosis (mini) gastric bypass (OAGB). There have been various studies comparing the outcomes of SG vs OAGB and this study aims to add our experience and compare one-year outcome data between SG and OAGB in a single UK bariatric centre.

Methods

A retrospective search of our database between June 2021 and August 2023 was performed to identify those patients undergoing either laparoscopic SG or OAGB. Initial and one-year follow-up data was collected including percentage total weight loss (%TWL), percentage excess body weight loss (%EBWL), incidence of post-operative reflux, remission of co-morbidities (diabetes), glycated haemoglobin (HbA1c) changes, operating time and post-operative complications.

Results

A total of 64 OAGB and 53 SG patients were identified in this time frame. Nineteen OAGB and 26 SG patients had one-year outcome data available and so were included in the final analysis. Pre-op BMI was significantly lower in the OAGB group (OAGB = 47.1, SG = 52.7, p<0.05). Initial age, rates of pre-operative gastro-oesophageal reflux symptoms and pre-operative diabetes were comparable. Regarding one-year outcomes, %EBWL was comparable, as was the length of stay, reduction in HbA1c and resolution of diabetes. Operating time was significantly shorter in the SG group (OAGB = 140 mins, SG = 111 mins, p<0.05). While the number of patients with post-operative complications was the same in both groups, two patients in the OAGB group suffered from ulcer disease with one requiring a return to theatre for this. No patients in the SG group suffered from ulcer disease. One OAGB patient required conversion to Roux-en-Y gastric bypass (RYGB) for reflux, while three SG patients required conversion to RYGB for resistant reflux.

Conclusion

Both OAGB and SG patients in our centre have comparable outcomes with regard to excess body weight loss and resolution of diabetes. SG was quicker to perform. OAGB may be associated with higher rates of ulceration while SG may be associated with higher rates of treatment-resistant reflux requiring conversion surgery. The literature has revealed greater weight loss and increased rates of diabetes resolution with OAGB. This along with our findings will be considered when counselling our patients on the bariatric procedures available to them.

## Introduction

Metabolic surgery has been proven to be a superior form of weight loss compared to non-operative measures for several years now [1]. Sleeve gastrectomy (SG), now a stand-alone procedure, was initially introduced as the first part of a two-stage operation, with the second stage either being a biliopancreatic diversion with duodenal switch or Roux-en-Y gastric bypass (RYGB) [1,2]. The procedure itself involves removal of the fundus and greater curvature of the stomach to create a smaller remnant stomach and has become the most popular bariatric procedure worldwide in terms of procedures performed [3,4].

One anastomosis gastric bypass (OAGB) or mini gastric bypass has also increased in popularity over recent years. OAGB involves creating a narrow gastric pouch and anastomosing it to the small bowel with a large proportion of jejunal bypass and is now the third most popular procedure for weight loss worldwide after SG and RYGB [5].

The outcomes of SG vs OAGB have been discussed over recent years. The aim of this report is to compare our experience and one-year outcome data from these two bariatric operations in a single UK bariatric centre.

This article was previously presented as a poster at the 2024 British Obesity & Metabolic Surgery Society (BOMSS) annual scientific meeting on 4th-5th June 2024.

## Materials and methods

Data collection

A retrospective search of our database from June 2021 to August 2023 was performed to identify patients undergoing either laparoscopic SG or laparoscopic OAGB in our centre (Walsall Manor Hospital, Walsall). Only patients undergoing fully laparoscopic procedures with evidence of one-year follow-up data were selected for further analysis. Re-do procedures were excluded. Each patient had undergone extensive discussions with members of the bariatric multidisciplinary team before being offered surgery.

Demographic data collected included gender, pre-operative weight, body mass index (BMI), pre-operative diabetes status, American Society of Anaesthesiologists (ASA) grade and pre-operative gastro-oesophageal reflux disease (GORD) symptoms. Diabetic patients were defined as those patients who had glycated haemoglobin (HbA1c) levels >48 mmol/mol.

Operative technique

Standardised approaches were used for both the laparoscopic OAGB and laparoscopic SG in our centre.

OAGB

After the creation of the gastric pouch using laparoscopic staplers with SEAMGUARD® (W. L. Gore & Associates, Flagstaff, Arizona), a 150cm biliopancreatic (BP) limb was formed and anastomosed to the gastric pouch at this point.

SG

The gastric sleeve was created using laparoscopic staplers with SEAMGUARD® against a standard 45-French orogastric tube.

Outcomes

Data on percentage total weight loss (%TWL), percentage excess body weight loss (%EBWL), incidence of post-operative gastro-oesophageal reflux, remission of diabetes, glycated haemoglobin (HbA1c) changes, length of stay, operating time and post-operative complications were compared between the two groups.

%TWL was calculated by dividing the total weight in kg lost by the patient’s initial weight and %EBWL was calculated by dividing the difference in pre- and post-op BMI by the difference between the pre-op BMI and 25 kg/m^2^ (which is the upper limit of a normal BMI) [6].

Statistical analysis

Statistical analysis was performed using Microsoft Excel (Microsoft Corporation, Redmond, USA). Continuous data are presented as mean ± standard deviation and analysed using a t-test. Categorical data are presented as numbers and percentages and analysed using a chi-squared test. Data was considered statistically significant when p<0.05.

## Results

A total of 117 patients (64 OAGB and 53 SG) were identified in this time frame. Nineteen OAGB and 26 SG patients had one-year follow-up outcome data available and so were analysed further. The one-year follow-up rate was therefore 38%.

The basic demographic data for the two groups are given in Table 1. Age and gender were comparable between the two groups, whereas the pre-operative weights and BMI were significantly lower in the OABG group. The prevalence of pre-operative GORD symptoms and diabetes was comparable. The median ASA grade was 3 in both groups.

**Table 1:** Demographics of the OAGB vs SG groups GORD: Gastro-oesophageal reflux disease; SG: sleeve gastrectomy; OABG: one anastomosis gastric bypass; ASA: American Society of Anaesthesiologists

Characteristic	OAGB (n=19)	SG (n=26)	p-value
Male	3/19 (15.8%)	8/26 (30.8%)	0.13
Mean age	47.1 (21-66)	46.7 (22-63)	0.46
Mean pre-op weight, kg (range)	134.8 ± 32 (98.6-198)	152.4 ± 35.7 (107.6-278)	0.048
Median pre-op weight (kg)	126	152.5	
Mean pre-op BMI, kg/m^2^ (range)	47.1 ± 9.75 (35.8-70.6)	52.7 ± 7.17 (41.9-66.0)	0.02
Median pre-op BMI (kg/m^2^)	47.0	53.6	
Pre-op GORD symptoms (%)	6/19 (31.6)	5/26 (19.2)	0.76
Pre-op diabetes (%)	4/19 (21.1%)	5 (19.2%)	0.74
Median pre-op ASA grade	3	3	

Table 2 shows outcome data for both groups. The mean post-op weight was non-significantly lower in the OAGB group. The percentage total weight loss (%TWL) and percentage excess body weight loss (%EBWL) were comparable in both groups although the absolute total weight loss was significantly greater in the SG group. Operating time was significantly shorter in the SG group, whereas the average length of stay was similar.

**Table 2:** Outcome data of OAGB vs SG groups GORD: Gastro-oesophageal reflux disease; SG: sleeve gastrectomy; OABG: one anastomosis gastric bypass; T2RF: type 2 respiratory failure; RYGB: Roux-en-Y gastric bypass

Outcome		OAGB (n=19)	SG (n=26)	p-value
Mean post-op weight, kg	Mean ± standard deviation	105.6 ± 31.1	112.9 ± 27.8	0.21
	Range	69.4-170	73-190	
Percentage excess body weight loss (%EBWL)	Mean ± standard deviation	50.2 ± 28.6	49.9 ± 19.5	0.49
	Range	-5.11 - +113.1	+15.5 - +94.7	
Percentage total weight loss (%TWL)	Mean ± standard deviation	21.8 ± 12.2	25.9 ± 8.45	0.09
	Range	-2.1 - +44.0	+7.94 - +44.7	
Total weight loss (kg)	Mean ± standard deviation	29.2 ± 16.9	39.5 ± 16.1	0.02
	Range	-2.6 - +54.9	+12.6 - +88	
Operating time (mins)	Mean ± standard deviation	139.7 ± 62.1	110.6 ± 36.5	0.027
	Range	82-276	44-181	
Length of stay (days)	Mean ± standard deviation	2.58 ± 1.61	2.42 ± 1.84	0.38
	Range	1-7	0-10	
Remission of DM (%)		3/4 (75)	3/5 (60)	0.32
Reduction in HbA1c (mmol/mol)	Mean ± standard deviation	4.28 ± 5.23	5.58 ± 13.1	0.35
	Range	-3 - +17	-11 - +58	
Post-op GORD symptoms (%)		5 (26.3)	9 (34.6)	0.29
Return to theatre within one year (excluding conversion surgery)		1 (5.26%) (repair of perforated ulcer in alimentary limb)	1 (3.85%) (repair of obstructed abdominal wall hernia)	1
Other post-op complications		4 (21.1%) (perforated ulcer in alimentary limb; marginal ulcer (non-perforated); T2RF; autoimmune hepatitis)	4 (15.4%) (Port site wound infection; pre-existing abdominal wall hernia becoming obstructed; dysphagia; peri-gastric haematoma)	1
Conversion to alternative bariatric operation		1 (5.26%) OAGB to RYGB for reflux	3 (11.5%) SG to RYGB (2 for reflux; 1 for persistent vomiting and reflux)	0.32

Both groups showed similar reductions in the pre and post-op HbA1c levels and in the proportion of diabetics who had their condition put into remission. The incidence of post-operative GORD symptoms was greater in the SG group although this difference was not significant.

Both groups also showed similar complication rates and rates of return to theatre. However, there was a greater proportion of post-operative ulceration in the OAGB group. Other complications requiring hospital admission in the OAGB group include type 2 respiratory failure (T2RF) and autoimmune hepatitis. Complications requiring admission in the SG group include obstruction of a pre-existing abdominal wall hernia (requiring operative management), dysphagia and a peri-gastric haematoma which were both managed conservatively. One OAGB patient required conversion to RYGB due to persistent reflux, while three SG patients required conversion to RYGB due to either reflux alone or treatment-resistant vomiting and reflux combined.

## Discussion

OABG being the newer procedure has meant that there is interest in its outcomes in comparison to SG. Our recent one-year experience shows that the percentage of excess body weight lost in the OAGB group is both considerable and comparable to that achieved in the SG group. There have been varying findings amongst the literature regarding this, with some suggesting that OAGB is associated with a greater %EBWL compared to SG especially in the first one to two years post-operatively [7]. Other studies however have suggested that SG leads to a greater rate of weight loss in the first two years post-operatively which then tails off to leave OAGB superior by five years post-op [8]. The two groups in our study were not matched for BMI with the SG group being classified as super-obese with their pre-op average BMI of 53.2 kg/m^2^, in comparison to the OAGB group being just obese with an average BMI of 47.1 kg/m^2^. Interestingly, some studies have suggested that OAGB is superior in terms of weight loss for this super-obese patient group [9,10]. Further patient group matching and longer term follow-up would be beneficial to establish where our practice fits into these findings.

Operation time was significantly shorter in the SG group compared to the OAGB group in our study. This seems to contrast with the literature which often suggests that there is either no difference between the two operation types [7] or that OAGB is associated with a shorter operation time [8,10]. There have been suggestions for this in the literature including more complex cases being preferred to undergo SG consequently increasing the average operating time. There may also be an association between the learning curve effect and increased experience of a procedure; this can help to increase the efficiency when performing it. OAGB is a newer technique in our centre which may, as a result, lead to an increased time to perform.

Similar rates of diabetes resolution were found in our study with around two-thirds of patients having their diabetes resolved after undergoing either OAGB or SG. OAGB has previously been shown to be significantly superior at resolving pre-existing diabetes in obese patients [11], largely due to both the restrictive and malabsorptive properties of the procedure [12-14]. This therefore may be an aspect to consider when selecting patients for each operation going forward.

The rate of post-operative reflux symptoms was higher in the SG group although not significantly. The literature also puts SG patients at higher risk of reflux symptoms with some suggesting that the lower GORD rates in OAGB may be due to its decreased intragastric pressure in relation to SG [7,15,16]. Further evaluation with more participants and longer term follow-up would help in establishing a true relationship in our local patient group. This is particularly important as three of our SG patients required a further revision procedure due to refractory GORD symptoms.

While still low, ulcer rates have been shown to be consistently higher in OAGB compared to SG [8,17]. Our OAGB ulcer rate was found to be 11% in this study. The range quoted in the literature is 0.65 - 4% and explanations for this higher rate compared to SG include an ischemic effect around the region of the anastomosis and increased rates of bile reflux seen with OAGB [17]. Ulceration typically manifests with bleeding or perforation with the former often managed with proton pump inhibitors or endoscopic treatment. Perforation on the other hand usually requires laparoscopic or open repair +/- omentoplasty [18]. One of the two ulcers seen in our OAGB group was perforated and required operative management highlighting the potential morbidity associated with this complication.

Limitations

Our study is mainly limited by the small sample size and its retrospective nature. Consequently, we aim to add to this study with more patients and a greater length of follow-up to strengthen the statistical power of our results. There was also some heterogeneity in the pre-op weights between the two groups requiring further patient group matching going forward.

## Conclusions

Both OAGB and SG patients in our centre have comparable outcomes with regard to percentage total and excess weight loss, resolution of diabetes and length of stay. SG was a quicker operation in our centre. While the rates of GORD may be higher in SG, the rates of ulceration may be increased in OAGB. Analysis of the literature has revealed greater weight loss over a longer time period in the OAGB group, with associated increased rates of diabetes resolution. Going forward, our findings will be considered when counselling our patients on the bariatric procedures available to them.
